# Realtime assessment of vascular occlusion and reperfusion in animal models of intraoperative imaging – a pilot study

**DOI:** 10.1515/iss-2023-0003

**Published:** 2024-03-26

**Authors:** Jayanth Kandukuri, Aseem Jain, Parag Karmarkar, Hrishikesh Gadagkar, Harold Aberman, Qihong Wang, Abhishek Rege

**Affiliations:** Vasoptic Medical, Inc., Columbia, MD, USA; Noble Life Sciences, Inc., Sykesville, MD, USA; Department of Pediatrics, University of Maryland School of Medicine, Baltimore, MD, USA

**Keywords:** blood flow imaging, real-time feedback, surgical technology, noninvasive monitoring, augmented reality visualization

## Abstract

**Objectives:**

Intraoperative monitoring of blood flow (BF) remains vital to guiding surgical decisions. Here, we report the use of SurgeON™ Blood Flow Monitor (BFM), a prototype system that attaches to surgical microscopes and implements laser speckle contrast imaging (LSCI) to noninvasively obtain and present vascular BF information in real-time within the microscope’s eyepiece.

**Methods:**

The ability of SurgeON BFM to monitor BF status during reversible vascular occlusion procedures was investigated in two large animal models: occlusion of saphenous veins in six NZW rabbit hindlimbs and clipping of middle cerebral artery (MCA) branches in four Dorset sheep brain hemispheres. SurgeON BFM acquired, presented, and stored LSCI-based blood flow velocity index (BFVi) data and performed indocyanine green video angiography (ICG-VA) for corroboration.

**Results:**

Stored BFVi data were analyzed for each phase: pre-occlusion (baseline), with the vessel occluded (occlusion), and after reversal of occlusion (re-perfusion). In saphenous veins, BFVi relative to baseline reduced to 5.2±3.7 % during occlusion and returned to 102.9±14.9 % during re-perfusion. Unlike ICG-VA, SurgeON BFM was able to monitor reduced BFVi and characterize re-perfusion robustly during five serial occlusion procedures conducted 2–5 min apart on the same vessel. Across four sheep MCA vessels, BFVi reduced to 18.6±7.7 % and returned to 120.1±27.8 % of baseline during occlusion and re-perfusion phases, respectively.

**Conclusions:**

SurgeON BFM can noninvasively monitor vascular occlusion status and provide intuitive visualization of BF information in real-time to an operating surgeon. This technology may find application in vascular, plastic, and neurovascular surgery.

## Introduction

Measuring intraoperative vascular blood flow through real-time systems remains a necessity across different subspecialties [1], [2], [3]. During cerebrovascular neurosurgery, monitoring cerebral blood flow remains crucial for both ensuring appropriate parenchymal perfusion throughout the intervention as well as assessing postsurgical tissue viability. Monitoring blood flow in vessels feeding the surgical site such as an aneurysm or arteriovenous malformation (AVM) is also important because these vessels are often temporarily occluded by placing surgical clips to reduce blood loss during surgery [4, 5]. Therefore, real-time assessment of both occlusion following clipping procedures and re-perfusion following reversal of occlusion provides much-needed confirmation [6, 7]. Failure to receive adequate perfusion may result in significant functional deficit while failure to confirm stabilized vascular flow status may lead to complications such as hemorrhages or stroke [8]. Within vascular surgery, measuring intraoperative blood flow is used to gauge graft patency and quickly evaluate ischemia [9], [10], [11]. Further, in gastrointestinal procedures, studies have shown that assessment of intraoperative blood flow can help identify anastomotic leakage, which remains a major complication of these procedures [12].

The need for intraoperative assessment of blood flow has led to the consideration of multiple techniques including macroscale imaging techniques such as intraoperative MRI (iMRI) and digital subtraction angiography (DSA), as well as local perfusion assessment techniques based on ultrasound and optical modalities. iMRI and DSA typically require expensive operating suites. Furthermore, their usage often leads to significant disruptions to the surgical procedure, leading to increased surgical durations. The radiation dose and reliance on contrast agents also limits its ability to be repeated multiple times during a single procedure. Ultrasound probes that leverage the Doppler phenomenon to estimate flow under the probe tip are often the standard of care for rapidly gathering real-time blood flow measurements. However, these probes have limited spatial resolution and their size prevents them from being able to visualize narrow areas within the vascular bed [13]. A study exploring the use of Doppler probes for evaluating aneurysm clippings reported significant shortcomings and inaccurate estimates [14]. Angiographic techniques such as ICG videoangiography (ICG-VA) image perfused vessels with high resolution, contrast, and accuracy. ICG-VA, through an assessment of the fluorescence signal, is able to confirm patency of blood vessels, unresolved leakage, and nonperfusion in regions such as stabilized aneurysms in the surgical field of view. While ICG-VA has advantages of using a nontoxic dye, which uses near infrared (NIR) light permitting its simultaneous use with other modalities, its long clearance time prevents continuous use during cases. Additionally, flow assessment using dye-based methods such as ICG-VA inherently have a small but disruptive effect to the surgical procedure that is associated with the dye injection process followed by visualizing perfusion on an external monitor. Nonetheless, ICG–VA is the current gold standard for intraoperative imaging of perfusion during various vascular procedures.

Laser speckle contrast imaging (LSCI), a noninvasive technique of imaging blood flow patterns in tissue, addresses some of the shortcomings of ICG–VA. LSCI is based on the principle that when tissue is photographed under coherent laser illumination, the scattered light produces random interference patterns called speckles [15, 16]. If there is orderly movement on this tissue surface such as blood flowing through vessels, a blurring effect is observed in the speckle pattern that depends on the exposure time of the camera. The extent of blurring can be quantified and has been shown to correlate to velocity of blood flow [17, 18]. Unlike previously described methods, LSCI does not involve injecting any dye forms or harmful radiation and still captures blood flow information at a high spatial resolution. Other advantages of LSCI include its ability to provide blood flow information rather than merely a binary perfusion status, permitting assessment of vascular and tissue status with greater nuance. The use of speckle-based methods of blood flow imaging during neurosurgical procedures [13, 19] and reconstructive vascular procedures [2, 20] has previously been explored in the clinic. In each case, laser speckle data were obtained intraoperatively and analyzed for insights on perfusion status in multiple regions of interest such as detection of infarct areas [21].

Here, we report on a novel speckle-based prototype imaging system called SurgeON™ Blood Flow Monitor (BFM) and results obtained from its investigational use in animal models of vascular surgery. The SurgeON BFM™ is different from previously described speckle-based imaging systems in its ability to be a modular attachment to surgical microscope allowing it to operate simultaneously, synchronously, and unobtrusively with the surgical microscope. This system can provide a real-time video feed of blood flow information to an operating surgeon within the microscope eyepiece itself through augmented reality. We previously demonstrated the preliminary feasibility of real-time imaging of blood flow in small animal models using a prior prototype of the SurgeON BFM [22]. The present study was aimed at the use of large animal models to demonstrate the feasibility of the modular-designed SurgeON BFM to observe blood flow in a surgical field of view that is comparable to the human case, an important step prior to clinical investigations.

## Materials and methods

### SurgeON blood flow monitor – system description

**Figure 1:**
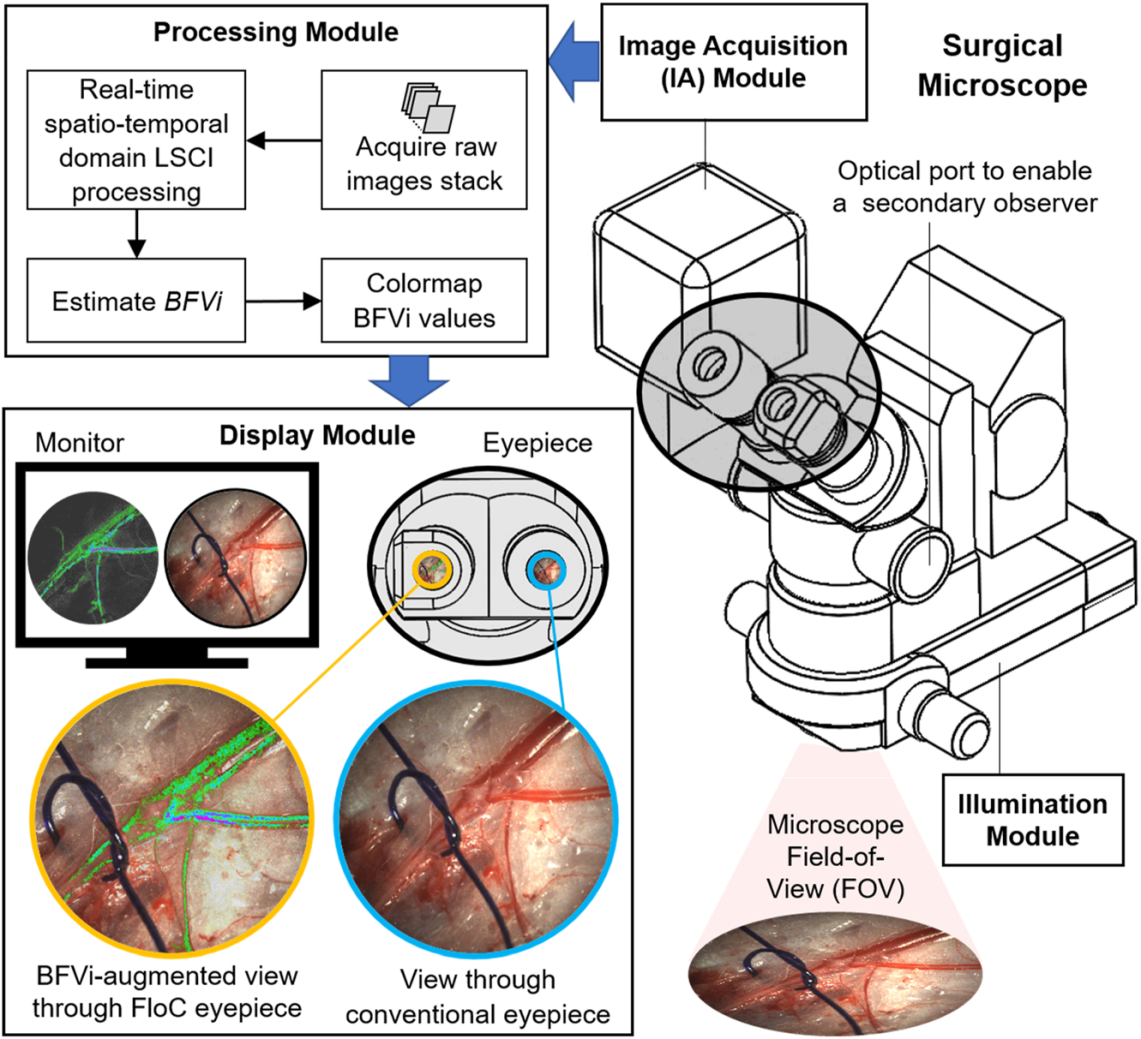
SurgeON system components and functional processes. Illumination and image acquisition modules attach in modular fashion to a surgical microscope, and acquired image data are processed in real-time to obtain blood flow velocity index (BFVi) estimates that are depicted in pseudo-color and displayed on a digital monitor or presented within the eyepiece overlaid on the live view of the surgical field.

The SurgeON BFM is an intraoperative blood flow monitoring prototype system with multiple improvements over its previously described precursor [22]. Distinctive features include its custom-developed augmented reality (AR) enabled eyepiece module (FloC) and its ability to implement both LSCI and ICG–VA modalities in tandem. The SurgeON BFM prototype used a refurbished Carl Zeiss OPMI MD operating microscope (OM) as its optical backbone. As shown in Figure 1, the SurgeON BFM used a Laser Illumination Module (LIM) that delivers, in conjunction with a lens assembly, a divergent near-infrared (NIR) laser beam with peak wavelength 785±10 nm that illuminates the entire surgical field of view (FOV). A temperature-regulated laser source prevented the laser from mode hopping. The Image Acquisition (IA) module, attached to the video port of the microscope, comprised of image formation optics and an aperture that leads to speckle production on a high-speed monochrome CMOS camera sensor. A second color CMOS camera with an NIR cutoff filter acquired live images of the surgical field of view in an optically equivalent position as the first camera. Image acquisition was triggered by a three-position foot pedal switch, which toggled between the modality (LSCI or ICG–VA) used, and controlled the ON/OFF function of the real-time visual feed. Speckle image data captured by the camera was either stored for offline use, or processed in real-time on a rolling basis to calculate speckle contrast (K) and estimate blood flow velocity index (BFVi), a numerical measure of relative blood flow at every pixel P_0_ at location (x_0_, y_0_) in frame n_0_, by solving Equations (1)–(3).

(1) K(P0)=σN(P0)μN(P0)

(2) $$N\left(P_{0}\right)=\left\{\begin{array}{c} P\left(x,y,n\right)\;s.t.\;\\ \left\|\left(x,y\right)-\left(x_{0},y_{0}\right)\right\|\leq 2\;px\;\\ \left|n-n_{0}\right|\leq 2\text{ frames} \end{array}\right\}$$

(3) $${K\left(P_{0}\right)}^{2}=\frac{1}{T\times \text{BFVI}}\left\{2-\frac{\left[1-\;\exp\left(-2T\times \text{BFVI}\right)\right]}{T\times \text{BFV}I}\right\}$$

where N(P_0_) represents a block of pixels in the spatio-temporal vicinity of P_0_ using which the standard deviation σ_N(P0)_ and mean µ_N(P0)_ of pixel intensities are computed, while T refers to the exposure time of the camera, which influences the sensitivity of BFVi measurements to underlying blood flow velocities. Spatio-temporal vicinity refers to a collection of 275 pixels around and including pixel P_0_ such that 5×5 pixels are chosen from each of 11 frames around its location and frame. To compute *K*_no_ for all pixels in the FOV at video rates, the ratio of standard deviation to mean of pixel intensities is computed simultaneously for all pixels at a given time (corresponding to frame *I*_no_) by maintaining two pixel arrays *S*1_no_ and *S*2_no_ for each 11 frame stack necessary for *K*_no_ computations and updating these arrays with every incoming frame *I*_no+5_ convolved with an averaging kernel *W* on a rolling first-in-first-out basis (see Equations (4)–(6)).

(4) $${S1}_{n_{0}}=\frac{1}{11}\overset{n_{0}+5}{\underset{n_{0}-5}{\sum }}I_{n_{0}}*W$$

(5) $${S2}_{n_{0}}=\frac{1}{11}\overset{n_{0}+5}{\underset{n_{0}-5}{\sum }}I_{n_{0}}^{2}*W$$

(6) $$K_{n_{0}}=\frac{1}{{S1}_{n_{0}}}\sqrt{{S2}_{n_{0}}-{S1}_{n_{0}}^{2}}$$

**Figure 2:**
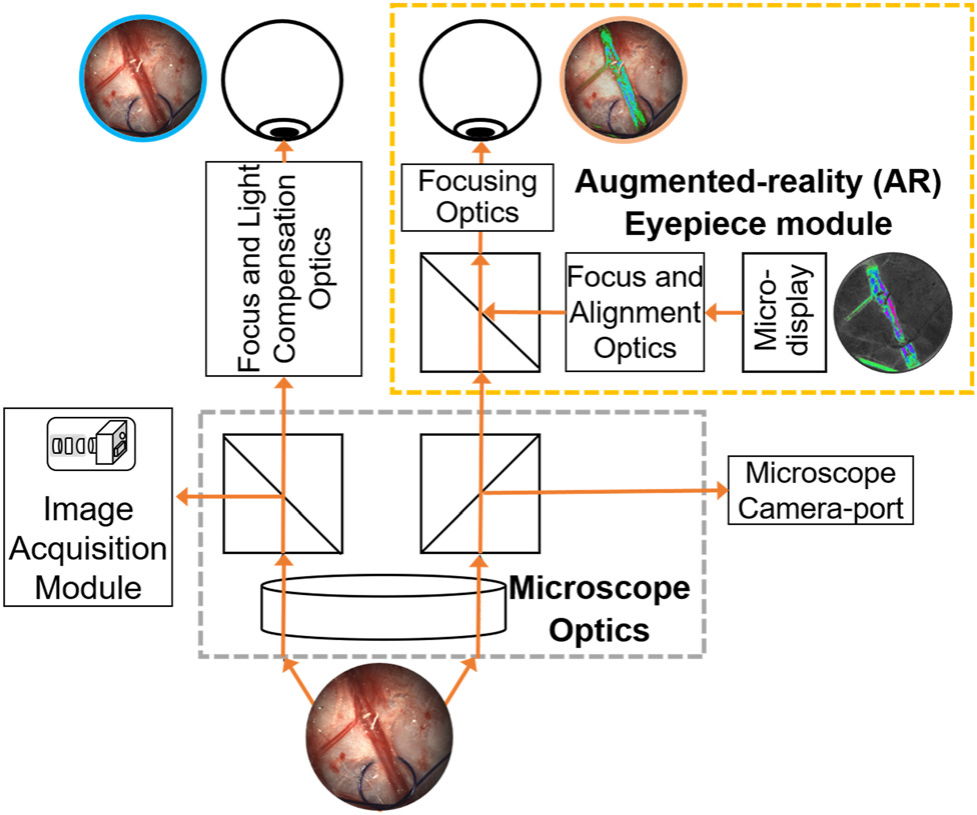
Schematic design of the optical elements in the custom eyepiece module that permits a real-time overlay of BFVi values depicted in pseudo-color on the live view of the surgical field. The eyepiece is custom-modified with a micro-display and optics to overlay blood flow information on the live view.

The use of GPU-based processing and precomputed lookup tables to convert K values to BFVi values and its pseudo-color rendition improved computational time, thereby providing a low-latency, real-time video feed of BFVi data. A pseudo-color representation of BFVi values at all pixels within the FOV (BFV image) was presented on an external display or on a micro-display located within a custom-built eyepiece called FloC™. A miniature beam splitter within FloC augmented the surgical site’s direct view with an overlay view of the micro-display, revealing blood flow information in real-time, as shown in Figure 2.

Implementation of the ICG–VA modality was achieved by selectively engaging an emission filter (long pass: 830 nm) using an electronic trigger-controlled filter slider in the imaging path. Additionally, by applying smoothing operations and intensity-based thresholding on the acquired data, the contrast-to-noise ratio of dye-containing vessels over background tissue was improved. All computations were implemented using MATLAB (Mathworks, MA, USA).

### Bench characterization of SurgeON BFM performance

The SurgeON BFM was calibrated following the installation of its various modules on the operating microscope to confirm uniform illumination of the field of view, alignment of the images captured by the two cameras, and alignment of the overlay. Standard imaging targets, white paper, and grid papers were used to confirm the field of view, spatial resolution, illumination uniformity, and the alignment of the images captured by the two cameras. An eyepiece camera was used to record the view available to a surgeon. It is also used to confirm the alignment and contrast quality of the augmented eyepiece view. To characterize the refresh rate and latency of the BFVi overlay output, a millisecond-sensitivity timer was imaged while the overlay was deliberately misaligned.

Prior to *in vivo* experimentation, we perfomed bench top flow testing to serve as a control for the data that we collected. We used an existing, calibrated flow pump to push dyed milk through a system of tubings that we could image using both LSCI and ICG. We found that we could achieve reliable, repeatable characterization of flow at different flow rates and various tubing diameter.

### Animal preparation and experimental methods

Proof of the feasibility of using the SurgeON BFM for intraoperative imaging of blood flow was demonstrated by employing the following two models: (a) rabbit model of saphenous vein occlusion and (b) sheep model of temporary cerebrovascular occlusion. We choose to test with rabbits to demonstrate proof of concept prior to performing the sheep model. All *in vivo* experiments in animal models were conducted at an accredited facility in compliance with the Institutional Animal Care and Use Committee.

#### Rabbit model of saphenous vein occlusion

The saphenous vein was specifically chosen for two reasons: one, because the saphenous vein is frequently used for various graft procedures, and two, because the saphenous vein can be reliably accessed without compromising nearby vasculature, making it useful as a proof of concept before more complicated dissections. This study was conducted on three New Zealand White rabbits with an approximate weight of 3 kg. Each rabbit was securely strapped to the operating table in the dorsal position and anesthetized via inhalation of 1.5 % isoflurane in oxygen. The veterinary surgeon then dissected the posterior hind leg and exposed the saphenous artery and vein for imaging. A surgical suture was looped around the saphenous vessels such that these vessels could be occluded by tightening the loop and the occlusion could be reversed by loosening the loop. Image data were acquired using the SurgeON BFM prior to vascular occlusion (baseline phase), rapidly after occlusion (occlusion phase), and then rapidly after reversing the occlusion (re-perfusion phase). ICG–VA was conducted during the occlusion phase for corroboration by injecting a bolus of ICG solution (5 mg powdered ICG per 10 mL of distilled water) intravenously immediately after occlusion while acquiring fluorescence data using SurgeON BFM in tandem with other imaging modalities. Subsequently, occlusion and reperfusion were carried out four additional times to assess the ability of SurgeON BFM to provide BFVi assessment unobtrusively at multiple times during a surgical case. Each occlusion phase lasted 30 s, and image data were acquired during the respective occlusion and re-perfusion phases. ICG–VA was also conducted during the second occlusion phase for corroboration and comparison.

#### Sheep model of cerebrovascular occlusion

Four adult Dorset sheep with weight in the range of 50–80 kg were subject to reversible vascular occlusion procedures outlined by Wells et al. [23]. The sheep were intubated and anesthetized via inhalation of 1.5 % isoflurane. An intravenous cannula was placed in the femoral vein for delivering fluids as needed throughout the surgical procedure. Each sheep was placed on the operating table in a rostral position with the head rotated laterally. The veterinary surgeon dissected through the dermis and muscular layers using electric cautery until the superior portion of the parietal bone was encountered. After clearing an area of approximately 10×10 cm using blunt dissection, a custom-modified drill was used to create a circular defect with an approximate diameter of 7.5 cm in the skull. The meninges were then carefully dissected until a significant branch of the middle cerebral artery (MCA) was visualized. A SugitaClip Titanium II mini clip (Mizuho America, Union City, CA) for temporary occlusion was placed on the exposed branch of the MCA to create a reversible occlusion and mimic temporary occlusions that are often carried out during cerebrovascular surgeries.

### Data acquisition

The SurgeON BFM computed BFVi images in real-time by implementing Equations (1)–(6) for each pixel of every acquired image frame, and the fully processed BFVi images data were stored along with live view images at 20 fps over the duration of the entire vessel occlusion experiment. Synchronously with the real-time dataset, images of the BFVi-augmented view of the surgical FOV were also recorded by an eyepiece camera at 18 fps. In addition, corresponding fluorescence (ICG-VA) data were also collected in some cases. For each of the following experiments, data using either or both modes were collected during each of the three phases: baseline, occlusion, and re-perfusion.

#### Rabbit saphenous vein occlusion model

Real-time datasets were obtained by SurgeON BFM over 40 s to include the entire reversible vessel occlusion procedure starting with baseline, through occlusion, reversal of occlusion, and stabilization of the reperfusion. ICG was injected immediately following the first vessel occlusion, and ICG-VA data were also collected simultaneously starting approximately 10 s after ICG administration or when fluorescence was detected in the FOV. After 2 to 5 min of flow stabilization following reversal of the first vessel occlusion, the vessel was occluded a second time, and a second real-time dataset was acquired to monitor the baseline, occlusion, and reperfusion phases associated with the second occlusion. ICG-VA data were recorded without injection of additional ICG because there was plenty of ICG already present in all vessels within the FOV. Three additional real-time datasets were obtained to assess blood flow during three additional occlusions of the same vessel to demonstrate repeated use of the LSCI modality.

#### Sheep model of cerebrovascular occlusion

Real-time datasets were acquired to monitor blood flow in the branch of the MCA over the entire occlusion experiment, that is, prior to clip placement (baseline), rapidly after clip placement (occlusion), and upon clip removal (re-perfusion).

### Data analysis

For analysis, stored real-time data were temporally spliced into 3 s segments that were exclusively associated with baseline, occlusion, and re-perfusion phases. For each vessel occluded, the mean flow was determined by averaging BFVi values of all pixels within a rectangular region overlaying a segment of a vessel just downstream of the occlusion site. Low-frequency noise resulting from motion artifact was reduced using adaptive thresholds. Mean BFVi values were estimated within the vessel segment of interest just downstream of the occlusion site by first spatially averaging all the BFVi values within the region of interest, then temporally averaging the obtained aggregates over all frames within the 3 s dataset, and finally averaging over the three repetitions during each phase. Estimated mean BFVi values were then normalized to the mean BFVi values to pre-occlusion levels for each surgical preparation. To assess intra-session repeatability, a mean BFVi was computed for each of three sequential nonoverlapping 1 s segments within each 3 s data set, and the coefficient of variation of these three mean BFVi values was computed. The fluorescence image frames obtained were postprocessed by moving window averaging across five frames (to remove random noise), followed by subtracting local minima from respective frames to finally obtain a scale-adjusted monochrome fluorescence dataset.

## Results

### Characterization of the SurgeON BFM specifications

When operated in the real-time mode, raw laser speckle image data were acquired by the NIR camera at 70 frames per second (fps) while BFV images were computed at video rates of 25 fps with a mean latency of 66 ms providing continuous and near-instantaneous visualization. The refresh rate drops to 20 fps when both live view and BFV images are streamed on the digital display. The SurgeON BFM can operate in either real-time or offline mode for postprocessing and analysis. The NIR and the visible camera can operate at two modes: 1200×1200 and 600×600 (with 2×2 binning), thus optimizing the spatial resolution for data acquisition and real-time visualization. The surgical microscope field-of-view (FOV) is 8 cm with a working distance of 50 cm (20 inches) with magnification 6.25× (maximum magnification 25×). The maximum laser power is 250 mW at the source but is substantially attenuated by the illumination optics and beam geometry to 10.6 mW at the working distance resulting in a mean irradiance of less than 1 mW/cm^2^ on the imaging target. In our rabbit hind limb model, the imaging FOV was 4 cm (at 12.5×) in diameter, while in the sheep brain model, the FOV for visualizing the exposed hemisphere was 2 cm (at 25×) in diameter.

### Rabbit model of saphenous vein occlusion

**Figure 3:**
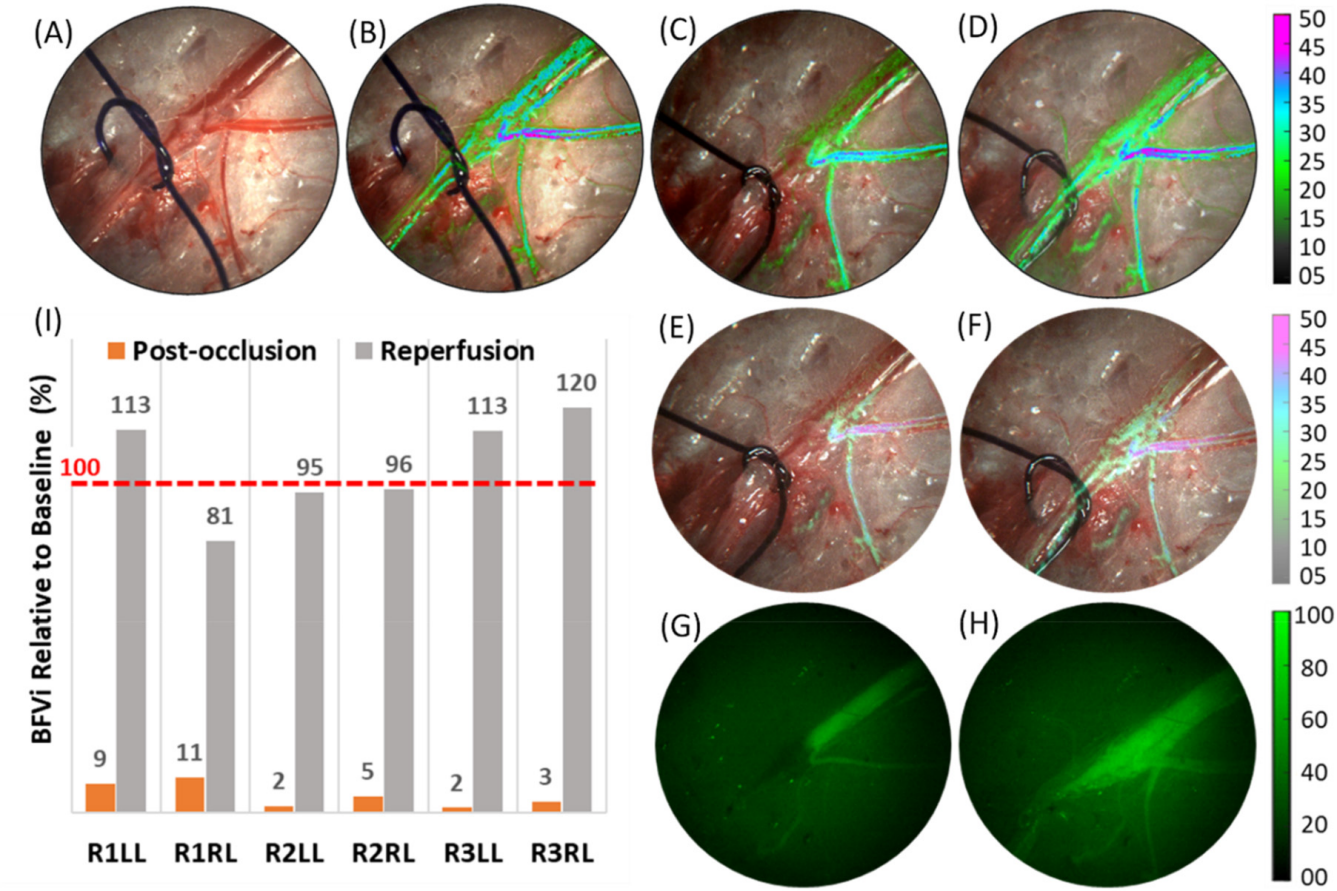
Surgical field of view (FOV) showing saphenous vessels of an NZW rabbit left hind limb. (A) Live view snapshot of the FOV; (B–D) overlay of blood flow velocity index (BFVi) image depicted in pseudo-color acquired during pre-occlusion, postocclusion, and re-perfusion phases, respectively, overlaid on the live view on a digital display, therefore, augmenting the information available during the surgery; (E and F) corresponding images of the same FOV with BFVi information overlaid on the live view as captured by an eye-piece camera reveal the two occlusion phases, respectively, in real-time to an operating surgeon without any workflow disruptions; (G and H) ICG videoangiography images obtained following ICG injection to confirm a vascular occlusion (G) and following ICG re-injection to assess a subsequent second vascular occlusion (H). (I) Results of BFVi-based confirmation of vascular occlusion and re-perfusion in six animal preparations.

As shown in Figure 3(A–F), SurgeON BFM was able to monitor blood flow in the saphenous vein of the rabbit hindlimb during baseline, occlusion, and re-perfusion phases. Blood flow information (BFVi, depicted in pseudo color) was overlaid on the live field of view and presented on both a digital display and within the FloC eyepiece. Occlusion of the saphenous vein was confirmed by lack of fluorescence in the vessel following ICG injection, as shown in Figure 3(G), and re-perfusion was confirmed by observed fluorescence in the vessel following reversal of occlusion, as shown in Figure 3(H). Across the six rabbit hindlimbs, mean BFVi was 18.2±6.0 a.u., 1.0±0.9 a.u., and 18.2±4.5 a.u. during pre-occlusion, occlusion, and re-perfusion phases, respectively (p<0.001, ANOVA), revealing a statistically significant drop in blood flow during in the occlusion phase. Figure 3(I) reveals BFVi during the occlusion and re-perfusion phases relative to baseline in each of the saphenous veins. When occluded, BFVi diminished to 5.2±3.7 % of pre-occlusion levels and returned to 102.9±14.9 % upon reversal of occlusion.

**Figure 4:**
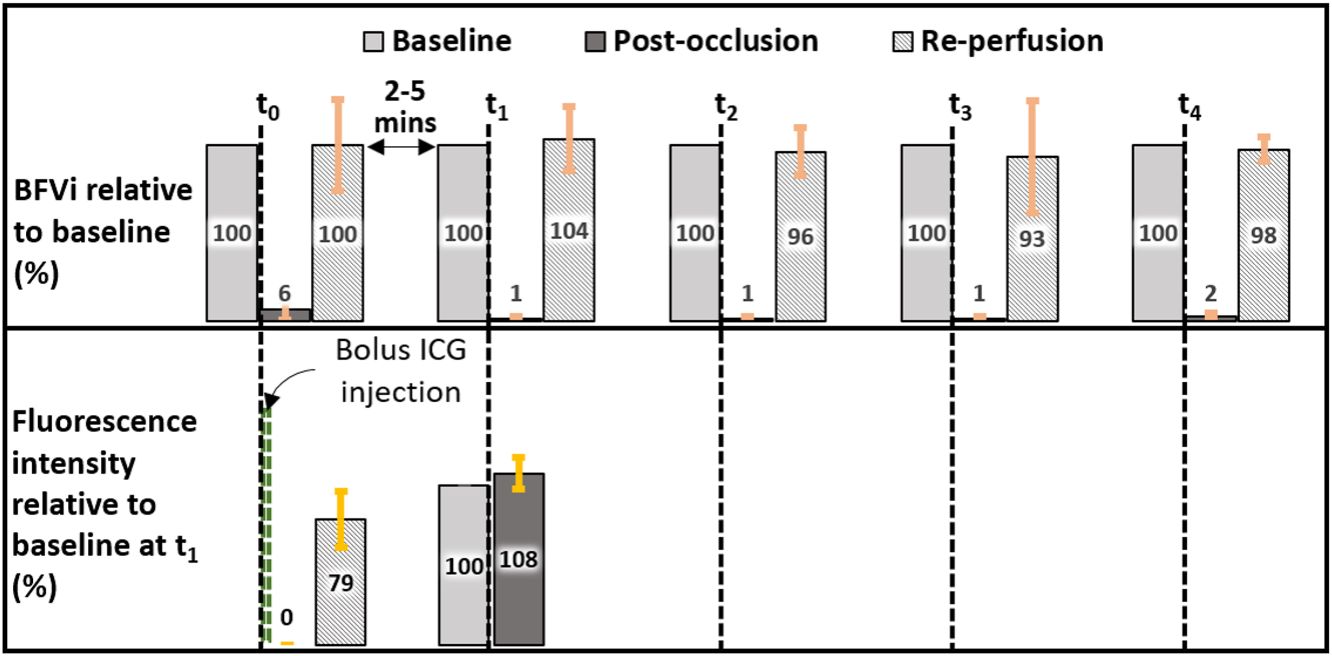
Event diagram depicting multiple reversible occlusions for comparison of LSCI-based and ICG-VA-based assessment. ICG was introduced after the first occlusion at time t_0_ and was not able to confirm occlusion at time t_1_ because the fluorescence intensity remained high. Error bars depict standard deviation across all six rabbit hindlimbs.

Figure 4 summarizes the performance of the LSCI modality while confirming the occluded or unoccluded status of saphenous veins multiple times during each surgical case. Relative to the baseline, BFVi values in the occluded vessels were 6.4±5.8 %, 1.2±1.4 %, 1.3±0.9 %, 1.5±0.9 %, and 2.3±2.1 % following each of five occlusion procedures across the six hindlimbs. In the re-perfusion phase, BFVi returned to 99.8±26.1 %, 103.6±18.6 %, 96.2±13.5 %, 93.3±32.2 %, and 97.6±7.3 % of the baseline levels. Mean fluorescence in the vessel was zero following the first occlusion, recorded at 80.1±17.0 a.u. soon after reversal of occlusion, and increased to 102.0±5.9 a.u. over the 2–5 min interval before the second occlusion. Mean fluorescence was 109.4±6.9 a.u. following the second occlusion.

### Reversible occlusion using aneurysm clip in the brain of a sheep model

**Figure 5:**
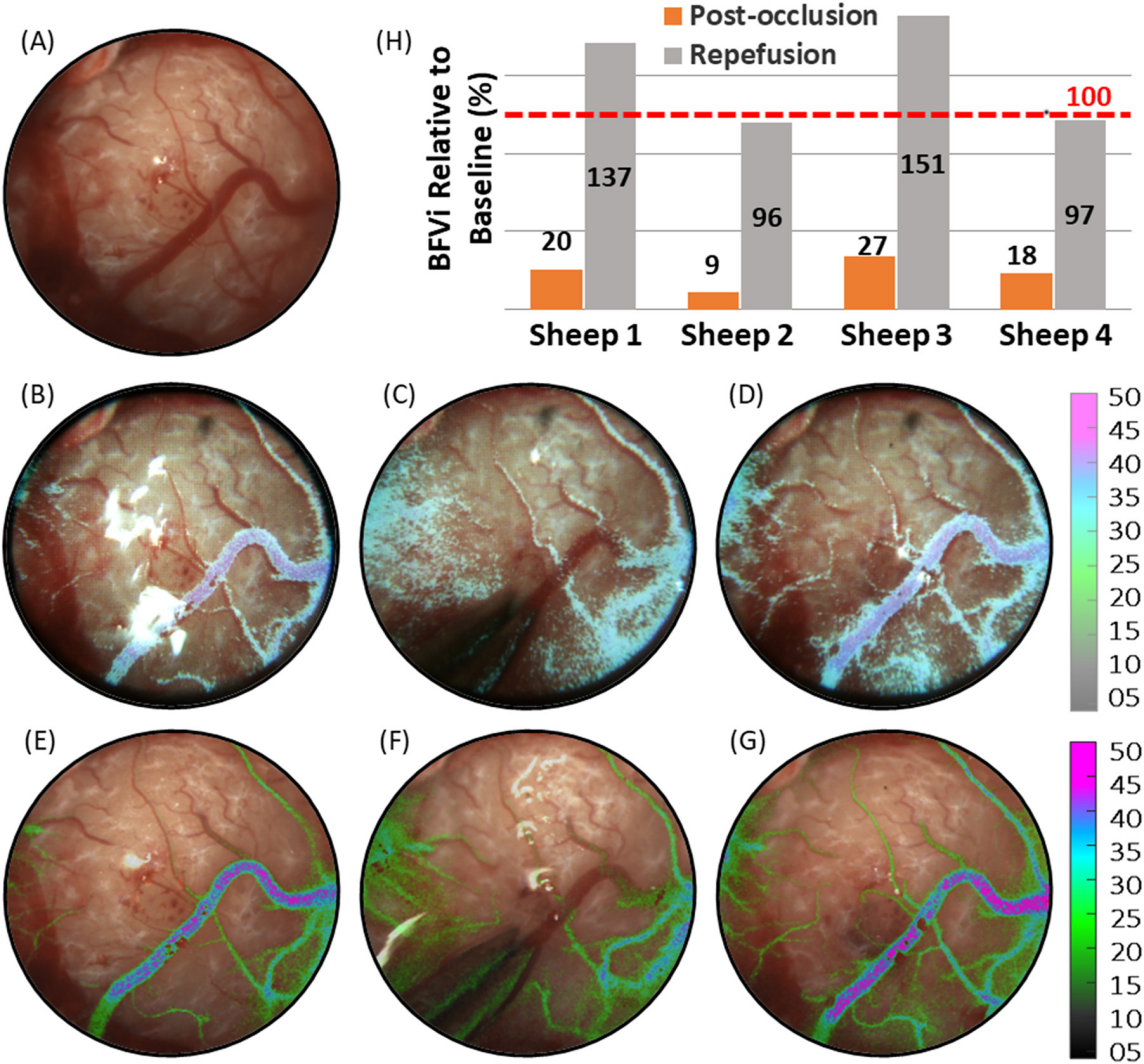
Surgical field of view (FOV) of the sheep brain during middle cerebral artery (MCA) branch occlusion with a surgical clip. The SurgeON system permitted visualization of vascular blood flow in sheep brain vessels in real-time directly in the eyepiece (A–C) and available digitally for viewing on an external display (D–F), thus enhancing the traditional view available to operating surgeons that is devoid of blood flow information (G). Blood flow velocity index (BFVi) is depicted in pseudo-color for real-time assessment of perfusion status in the surgical field during baseline conditions (A and D), immediately following clipping (B and E) and following unclipping or reversal of occlusion to confirm re-perfusion (C and F). (H) Assessment of BFVi relative to the baseline (pre-occlusion levels depicted by red dashed line) provides a quantitative assessment of occlusion status (orange) and the reperfusion status (gray) in the MCA branch in each of the four hemispheres (HS1–HS4) in total of four sheep.

Across the four occlusion procedures, mean BFVi was 10.2±0.8  a.u., 1.9±0.7 a.u., and 12.2±2.2 a.u. during the baseline, occlusion, and re-perfusion phases, respectively (ANOVA, p<0.001), revealing a significant difference between the occluded and unoccluded states as shown in Figure 5. On average, during the occlusion phase, BFVi reduced to 18.6±7.7 % of pre-occlusion levels and returned to 120.1±27.8 % upon reversal of occlusion.

### Repeatability of measurements

**Figure 6:**
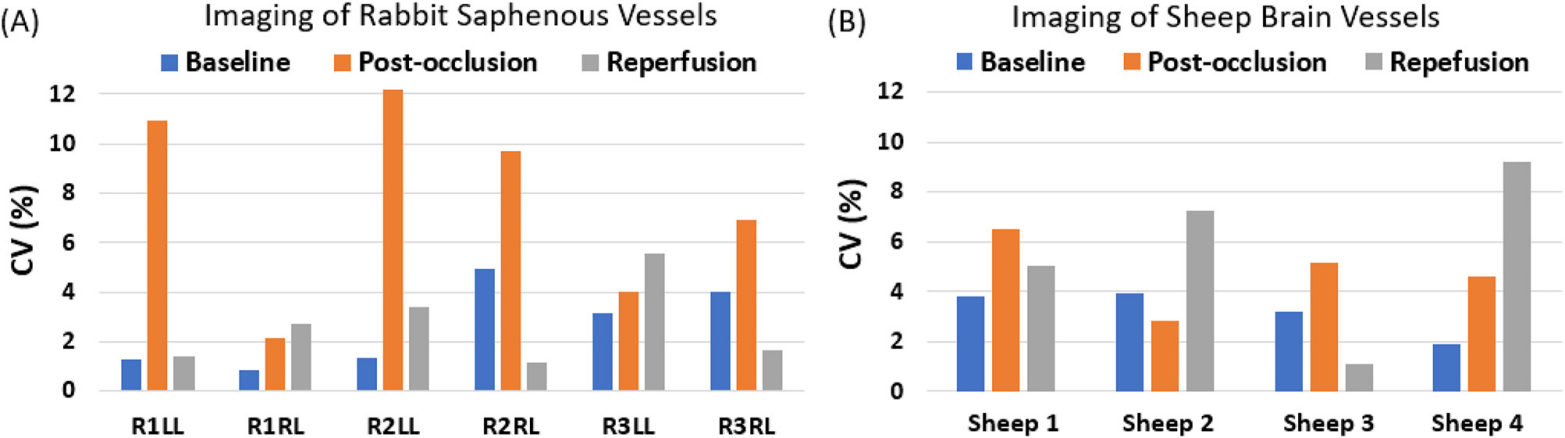
Intrasession variability in blood flow measurements. The intrasession coefficient of variation (CV) in BFVi measurement observed in (A) saphenous vessels undergoing occlusion procedures in the rabbit hindlimb and (B) branch of the middle cerebral artery undergoing occlusion procedures in the sheep brain.

As reported in Figure 6, the mean coefficient of variation (CV) of BFVi measurements from the saphenous vein segments in the rabbit hindlimb were determined to be 2.6±1.7 %, 7.7±4.0 %, and 2.6±1.7 % for baseline, occlusion, and re-perfusion phases, respectively. Similarly, CV of BFVi measurements in the branch of the MCA that was occluded was determined to be 3.2±0.9 %, 4.8±1.5 %, and 5.6±3.5 % for baseline, occlusion, and re-perfusion phases, respectively.

## Discussion

The current prototype of the SurgeON BFM was developed to be attached externally to a standard operating microscope without requiring any changes to the microscope components. The image acquisition module attaches to the video port of the microscope using standard mounts. The use of NIR illumination for LSCI does not interfere with the course of the surgery that is often carried out under bright broadband illumination. Light in the NIR spectrum also does not pose any risk of photochemical toxicity to the exposed tissue, thus limiting the potential risks to only thermal consideration, the thresholds for which are generous [24]. The illumination is designed to be safe also through the choice of a diverging beam geometry as it exits the illumination module mounted on the microscope, thereby limiting the irradiance at the tissue being operated upon as well as on the anterior and posterior segments of the eyes of one or more operators in close proximity to the surgical site [25]. The FloC eyepiece is the only part of the SurgeON BFM that requires substitution or modification of the microscope components and is an optional feature for the operating surgeon to obtain a live feed of blood flow information into the eyepiece.

Real-time implementation of LSCI is enabled by a combination of spatial and temporal relationships in speckle patterns to balance not only the spatial and temporal resolution with which blood flow information from the target tissue is obtained but also to manage the computation time required to generate and display BFVi values [26]. Further improvement in imaging specifications is possible as newer cameras deliver improved resolutions at faster frame rates and with improvement in processor speeds via GPU or parallel computing technologies. Our achieved latency of 66 ms and a refresh rate of 20 fps meet the minimum requirements for the typical persistence of human vision for smooth, continuous real-time live view of blood flow information. LSCI relies on the extent of speckle blurring over the camera exposure time allowing users to modify camera exposure time as a parameter to either adjust the sensitivity of blood flow monitoring or increase the dynamic range of blood flow measurement [17, 27, 28]. In the BFVi overlay images of Figures 3 and 4, parameters were adjusted to preferentially visualize blood flow changes in the large vessel within the FOV, while ignoring the nutritive perfusion provided by small caliber vessels in favor of maintaining a cleaner FOV while still confirming occluded or nonoccluded status. Use of multiple exposures for robust blood flow monitoring has been previously demonstrated in the intraoperative setting but not in a real-time, low-latency manner [29]. LSCI is susceptible to motion artifact and, therefore, tissue motion may pose challenges for imaging during surgical cases. The high frame-rate with which SurgeON BFM acquires and processes raw data mitigates the effect of low frequency motion to a certain extent. Additional motion compensation mechanisms may be applied intraoperatively but not during real-time capture to obtain more accurate estimates of blood flow [30, 31]. While motion artifact may reduce the accuracy of blood flow measurement to a small extent, the impact is not expected to be substantial enough to impact the goal of monitoring occlusion or perfusion status of blood vessels. SurgeON BFM’s ability to image blood flow during a surgical case may also be influenced by factors such as pooling of blood or lack of a direct line of sight access to deeper but exposed structures. However, these are challenges common to most optical imaging modalities. Because the LSCI signal depends on blood motion, SurgeON BFM may have a better chance of resolving blood flow under a static pool of blood or clot than other angiographic techniques that will result in a contaminated field because of dye extravasation.

Blood flow data revealed by LSCI is not absolute and, therefore, BFVi is expressed in arbitrary units. BFVi captures the effect of both blood speed as well as vessel diameter, as red blood cell flux within the entire three-dimensional depth of field contributes to the speckle signal at any two-dimensional pixel [22, 32]. This may be considered both a limitation and an advantage because volumetric flow of blood is more relevant to physiology and function and provide more useful information to a surgeon than an assessment of blood speed. Further, baseline is relatively easy to establish during a surgical case, following which SurgeON BFM can provide a quantitative assessment of blood flow status relative to baseline, as demonstrated by our current *in vivo* results.

Large vessels often pulsate visibly when perfused making it easy to confirm occlusion status without the use of microscopes. However, it is difficult to perceive perfusion in smaller caliber vessels and represents an avenue where imaging technologies can assist. The rabbit saphenous veins imaged by the SurgeON BFM had diameters in the range of 1.0–1.1 mm and represent the approximate caliber where imaging can begin to add insights. The purpose of utilizing a saphenous vein model was to demonstrate successful imaging of blood flow within a surgically exposed vessel that is often used for graft procedures [33, 34]. The saphenous vein is commonly harvested and used as a bypass graft in various procedures including coronary bypass. The ability of the SurgeON BFM to monitor blood flow in a segment of the saphenous vein, even though the saphenous vein was in its native and not graft configuration, suggests that blood flow monitoring could provide a way to assess graft patency [35, 36].

**Figure 7:**
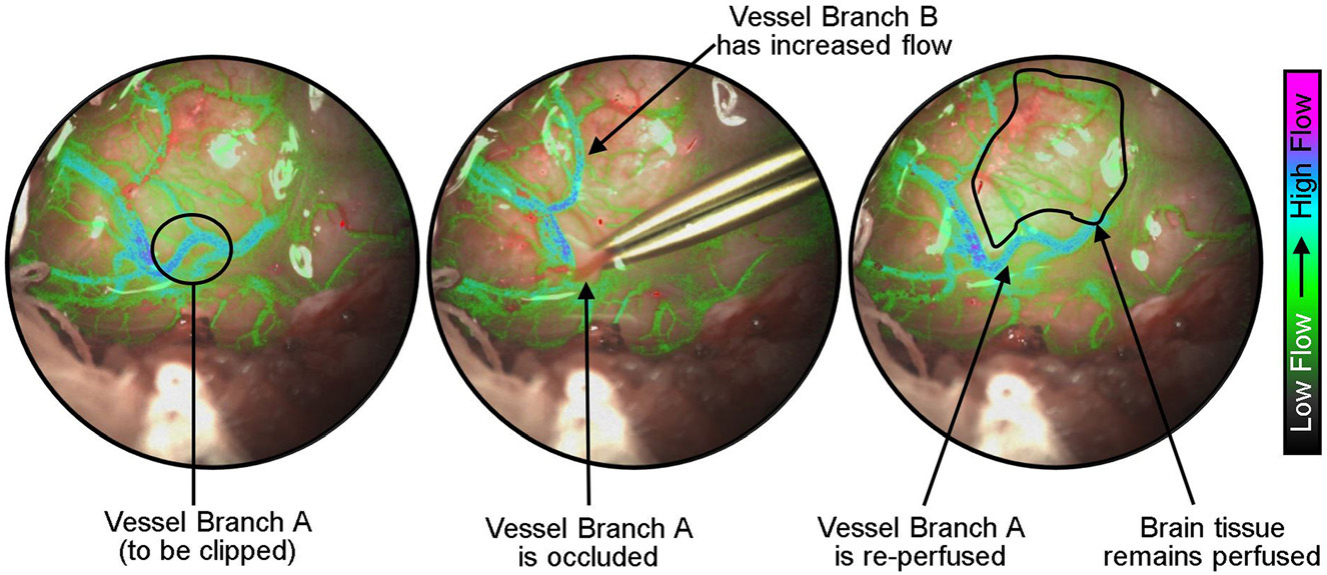
Reversible clipping of a brain vessel. Laser speckle contrast imaging permits real-time assessment of blood flow in not just the vessel undergoing occlusion but also other major and minor vessels within the field of view, thus keeping the surgeon informed of the perfusion status of the brain tissue.

The purpose of utilizing a reversible occlusion model of a significant brain vessel was to demonstrate the usefulness of the SurgeON technology for cerebrovascular neurosurgery. Temporary clips are often placed on significant vessels during neurosurgery to prevent bleeds during the procedures. However, clipping must also be balanced with the risk of sustained underperfusion of various regions of the brain. Therefore, a real-time blood flow monitoring technology may provide the neurosurgeon useful feedback. There is also a significant need to confirm that blood flow has been adequately restored in proximal and distal regions following the removal of the clip. In our experiment, we were able to adequately confirm that the branch of the sheep MCA was occluded by the placement of the Sugita clip and then confirm that flow had returned to mostly baseline levels upon the removal of the clip. As shown in Figure 7, because of the full-field nature of LSCI, it becomes possible to monitor perfusion status of not just the vessel underdoing reversible occlusion but also of other proximal vessels including the nutritive perfusion of the brain tissue within the field of view. This attribute provides the surgeon with holistic information with the click of a button and distinguishes this approach from perfusion assessment provided by probes such as an ultrasonic flow probe or a laser Doppler flowmeter [4, 37]. Additionally, availability of blood flow information in the eyepiece itself implies that the surgeon can continue performing the surgery without the need to take a break for monitoring purposes and, therefore, potentially use the modality for optimization of clip placement.

As described above, multiple authors have compared a contrasted ICG-VA and LSCI methods. ICG-VA can typically attain greater signal to noise ratio compared to LSCI. However, an important advantage of LSCI over dye-based angiography such as ICG-VA is the ability to obtain blood flow information on an on-demand basis: a tool that is vital for intraoperative blood flow management. As we observed in Figures 3(G and H) and 4, ICG-VA had not cleared sufficiently leading to false positives while being used a second time to confirm occlusion within a few minutes of a prior use. Clinically, ICG-VA has been used for multiple reperfusion; however, a significant amount of time is required between each perfusion. However, LSCI was able to report a reduction in blood flow in real-time confirming occlusion, and unlike ICG-VA also confirm that blood flow has returned to levels comparable to pre-occlusion levels. Anecdotally, during the reversible occlusion experiments, there were two instances when LSCI was able to inform the veterinary surgeon performing the occlusions that the vessel may not have been completely occluded, and that the maneuver needed to be revisited.

While the two vascular occlusion models employed in this study were associated with significant vessels, LSCI can be used to monitor other vessels of varying calibers as well, including microvessels that characterize tissue perfusion [38], [39], [40]. Prior research carried out by our group has already shown the ability of LSCI to reveal blood flow patterns and microvascular morphology associated with angiogenesis and also to assess the ischemic insult to cortical regions following cauterization of a branch of the middle cerebral artery [22, 41]. The spatial resolution and field of view of blood flow visualization is determined by the magnification status of the microscope. Pseudo-color representation of the blood flow velocities can be adjusted and calibrated to provide a desired visual contrast and flow-sensitivity. However, blood flow in a significantly large vessel has a different order of magnitude than capillary perfusion feeding a tissue. The SurgeON BFM technology has a dynamic range that is higher than most techniques and is adjustable by setting an appropriate exposure time for data acquisition but may still need optimization for either larger flows or smaller flows during real-time use where multi-exposure image acquisition is not an option [17].

The results from this pilot study in large animal models are promising, but additional development and optimization is necessary for clinical translation. Such optimizations include, but are not limited to, modification of SurgeON technology for seamless compatibility with current surgical microscopes, integration of the SurgeON technology with the sterile workflow of the operating room, and appropriate training of the operating surgeons and supporting staff.

## Conclusions

We have successfully demonstrated in two animal models of reversible vascular occlusion that SurgeON BFM is able to leverage the LSCI modality to noninvasively present blood flow information to an operating surgeon in real-time, quantitatively compare it to baseline blood flow, and thus monitor occluded and non-occluded status of vessels.

## Supplementary Material

Supplementary Material
