# Supplementary material for: Realtime assessment of vascular occlusion and reperfusion in animal models of intraoperative imaging – a pilot study
Source: Innov Surg Sci. 2024 Mar 26;9(1):25–35. doi: 10.1515/iss-2023-0003 (PMC11138401; doi:10.1515/iss-2023-0003)
Supplement: Supplementary file 1 — Supplementary Material [file j_iss-2023-0003_suppl_001.pdf]

Reviewer Assessment

R. Abhishek et al.: REALTIME ASSESSMENT OF VASCULAR OCCLUSION AND REPERFUSION IN ANIMAL MODELS OF INTRAOPERATIVE IMAGING: PILOT STUDY

Reviewers' Comments to Original Submission

Reviewer 1: anonymous

Date received: 16-Feb-2023
Reviewer recommendation: **Return to author for major modifications**
Reviewer overall scoring: **High**

Comments to author:
In this study the value and accuracy of LSCI technique is compared to ICG in 2 different animal models. Bote include temporary and repeated occlusion of blood vessels: GSV (rabbit) and MCA (sheep). The study per se is conducted scientifically sound, the question to be answered is innovative and of practical relevance and the manuscript is well-written.
However, there are 2 basic critical points that need to be addressed:
a) why did the authors use a temporarily occluded GSV (rabbit)? It seems not clear, in which clinical scenario the temporary occlusion of a vein is relevant, nor its monitoring (by whatever technique). The authors should explicitly refer and explain this clinical need, if existent. Since ICG is typically only used (and recommended) to monitor arterial perfusion it is not surprising that when assessing flow through in a vein, ICG underperforms against LSCI due to persisten dye in the venous system. Again the question arises, why this model/scenario had been chosen for comparison in the first place
b) why has patentcy/flow through/perfusion within single vessels been chosen as the main (and only) parameter? From the reviewers point of view, the rationale for using ICG or LSCI (or any other perfusion-monitoring/imaging technique) usually is not only used to assess blood flow in a single vessel (as e. g. the pedicle vessels in flap transplantation), but also of the tissue, which is nourished by particular vessel (is it transplantete kidney tissue, flaps, brain tissue after clipping of aneurysms, etc....). In this context, dynamic, repeated and even "real-time" imaging by using LSCI could be beneficial or even superior to ICG, but has not been investigated here. Why not?

Minor points:
The disadvantage of ICG as postulated on "page 6 of 30" at the end of the first paragraph can not be confirmed, since it does not really pose "workflow challenges", nor doe it cause the need fot additional ressources (which? the camera = yes, but this applies for LSCI as well...) or personnel (these procedures will rarely be peformed without an anesthetist anyhow) nor does ist significantly proloing operating time- while it still has some drawbacks as pointed out in the manuscript elsewhere.
Furthermore there should be more graphic illustrations of a) the saphenous vein occlusion model, not only the MCA-Model and b) also of the ICG experiments

Authors' Response to Reviewer Comments

Date received: 05-May-2023

The authors thank the editorial and review panel for finding our manuscript scientifically sound, innovative, and practically relevant, and for their thoughtful critique that requires a revision. Below is our response to each of these comments, along with a since-strengthened revised manuscript. Revisions are highlighted in gray in the manuscript text for ease of review.

Reviewer Comment #1. Why did the authors use a temporarily occluded GSV (rabbit)? It seems not clear, in which clinical scenario the temporary occlusion of a vein is relevant, nor its monitoring (by whatever technique). The authors should explicitly refer and explain this clinical need, if existent. Since ICG is typically only used (and recommended) to monitor arterial perfusion it is not surprising that when assessing flow through in a vein, ICG underperforms against LSCI due

to persisten(t) dye in the venous system. Again, the question arises, why this model/scenario had been chosen for comparison in the first place.
Response: Authors thank the reviewer for pointing out this need for clarification. We had previously mentioned only briefly the relevance of this rabbit saphenous vein model in the discussion section of the manuscript, but have now elaborated more on the model's suitability and interpretation in the revised manuscript (Lines 166 – 170, Lines 353 – 361). As an explanation to the reviewer - the purpose of the model was purely to demonstrate the ability of our technology to monitor blood flow through a vessel that is routinely used as a graft in bypass procedures. With this is mind, the saphenous vein was specifically chosen for two reasons: one, because the saphenous vein is frequently used for various graft procedures, and two, because the saphenous vein can be reliably accessed without compromising nearby vasculature making it useful as a proof of concept before more complicated dissections. As a first experiment, we found it reasonable to demonstrate this ability with the vessel in its native state with blood flowing through the vessel lumen. Occlusion and release of occlusion was a way to demonstrate the ability of our technology to detect flow (patency) or no flow (non-patency) through it. We determined that performing additional surgical procedures such as suturing the graft in an animal model (as would be needed to mimic an actual bypass experiment) would not add much value to a demonstration of the blood flow monitoring ability of our technology. We envision investigating graft patency directly in the clinical operating room during a human surgical case. Surely, ICG highlights arteries more rapidly and with higher contrast than veins, however, as stated, saphenous vein was chosen as a model that possesses the characteristics of a vessel that is used as an in vivo graft conduit. Also, LSCI can image blood flow in both arteries and veins with high contrast, and offer useful information intraoperatively.

Reviewer Comment #2. Why has patentcy/flow through/perfusion within single vessels been chosen as the main (and only) parameter? From the reviewers point of view, the rationale for using ICG or LSCI (or any other perfusion-monitoring/imaging technique) usually is not only used to assess blood flow in a single vessel (as e. g. the pedicle vessels in flap transplantation), but also of the tissue, which is nourished by particular vessel (is it transplantete kidney tissue, flaps, brain tissue after clipping of aneurysms, etc....). In this context, dynamic, repeated and even "real-time" imaging by using LSCI could be beneficial or even superior to ICG, but has not been investigated here. Why not?
Response: Authors thank the reviewer for this suggestion, and share the reviewer's enthusiasm for an expanded role of LSCI in assessing tissue perfusion. We agree that LSCI can offer significant advantages over ICG angiography by providing a real-time assessment of tissue perfusion. However, the ability of LSCI to obtain tissue perfusion information has been routinely described in literature for various research applications,1-3 including by our group.4, 5 In particular, we have previously published results obtained by using LSCI-based SurgeON technology to measure the ischemic insult caused on cortical regions by middle cerebral artery cauterization.6 Figure 7 of our manuscript conveys this benefit and application. However, we have chosen here to focus our report on demonstrating our ability to monitor vascular blood flow in real time in vessels that may be candidates for graft procedures or reversible occlusion procedures. Another reason is that blood flow in a significantly large vessel has a different order of magnitude than capillary perfusion feeding a tissue. The SurgeON BFM technology that utilizes LSCI has a dynamic range that is larger than many techniques and adjustable by setting an appropriate exposure time for data acquisition, but may still need optimization for either larger flows or smaller flows during real-time use where multi-exposure image acquisition is not an option.7 Our system was optimized for visualization of higher blood flows with greater sensitivity. Regardless, this is a great suggestion, and we have included a discussion of this aspect in the Discussion section of the revised manuscript (Lines 394 – 407).

Reviewer Minor Comment #1. The disadvantage of ICG as postulated on "page 6 of 30" at the end of the first paragraph cannot be confirmed, since it does not really pose "workflow challenges", nor doe(s) it cause the need fo(r) additional resources (which? the camera = yes, but this applies for LSCI as well...) or personnel (these procedures will rarely be performed without an anesthetist anyhow) nor does it significantly prolong operating time- while it still has some drawbacks as pointed out in the manuscript elsewhere.
Response: We thank the reviewer for pointing this out, and have rephrased the disadvantage stated on Lines 66 – 70.

Reviewer Minor Comment #2. Furthermore there should be more graphic illustrations of a) the saphenous vein occlusion model, not only the MCA-Model and b) also of the ICG experiments
Response: The authors would like to point out that Figure 4 graphically illustrates the saphenous vein occlusion experiment including ICG injection.
We hope that the above-mentioned response and concomitant changes to the manuscript adequately address the reviewer's concerns and that the revised manuscript is stronger and will be considered for publication.

References used in the Response
1. Mahe G, Humeau-Heurtier A, Durand S, Leftheriotis G, and Abraham P, "Assessment of skin microvascular function and dysfunction with laser speckle contrast imaging," Circulation. Cardiovascular imaging 5, 155-163 (2012).
2. Boas DA and Dunn AK, "Laser speckle contrast imaging in biomedical optics," J Biomed Opt 15, 011109-011112 (2010).
3. Heeman W, Maassen H, Calon J, van Goor H, Leuvenink H, van Dam G, and Boerma EC, "Real-time visualization of renal microperfusion using laser speckle contrast imaging," Journal of biomedical optics 26, 056004 (2021).
4. Rege A, Thakor NV, Rhie K, and Pathak AP, "In vivo laser speckle imaging reveals microvascular remodeling and hemodynamic changes during wound healing angiogenesis," Angiogenesis 15, 87-98 (2012).
5. Senarathna J, Rege A, Li N, and Thakor NV, "Laser Speckle Contrast Imaging: theory, instrumentation and applications," IEEE reviews in biomedical engineering 6, 99-110 (2013).
6. Mangraviti A, Volpin F, Cha J, Cunningham SI, Raje K, Brooke MJ, Brem H, Olivi A, Huang J, Tyler BM, and Rege A, "Intraoperative Laser Speckle Contrast Imaging For Real-Time Visualization of Cerebral Blood Flow in Cerebrovascular Surgery: Results From Pre-Clinical Studies," Sci Rep 10, 7614 (2020).
7. Rege A, Murari K, Seifert A, Pathak AP, and Thakor NV, "Multi exposure laser speckle contrast imaging of the angiogenic microenvironment," J Biomed Opt 16, 05600601-05600610 (2011).

Reviewers' Comments to Revised Submission

Reviewer 1: anonymous

Date received: 09-05-2023
Reviewer recommendation: **Accept in present form**
Reviewer overall scoring: **High**

Comments to author:

Thanks for addressing the critical points in a sufficient manner; the manuscript qualifies for being published in its present form.

Reviewer 2: anonymous

Date received: 14-09-2023
Reviewer recommendation: **Return to author for minor modifications**
Reviewer overall scoring: **High**

Comments to author:

I have read with great interest the article "Real time assessment of vascular occlusion and reperfusion in animal models of intraoperative imaging" which deals with the establishment of "SurgeON BFN" in a large animal model. The colleagues have already been able to publish their preliminary data at a high level (Sci Rep 2020) and therefore have visible expertise in laser speckle contrast imaging (LSCI). The clinical need for dynamic methods to assess organ perfusion in real time is unquestionable. The advantages of LSCI compared to ICG and conventional Doppler diagnostics have been demonstrated and discussed. The graphs are clear and facilitate the understanding of the study. As already criticized by the pre-reviewers, the Rabbit model is not ideal for the application of LSCI especially in comparison to ICG especially since the possibilities for the application of LSCI (transplantation, intestinal anastomoses etc.) seem almost unlimited.
Overall, this is a pilot study which should perhaps be mentioned in the title, since the results of the study are limited and above all the feasibility is demonstrated. Furthermore, the colleagues should mention in the discussion which concrete further steps are necessary to make LSCI clinically applicable.

Authors’ Response to Reviewer Comments on Revised Submission

Date received: 15-09-2023

The authors thank the editorial and review panel for finding our manuscript scientifically sound, innovative, and practically relevant, and for their thoughtful critique that requires a revision. Below is our response to each of these comments, along with a since-strengthened revised manuscript. Revisions are highlighted in gray in the manuscript text for ease of review.

Reviewer Comment #1. Thanks for addressing the critical points in a sufficient manner; the manuscript qualifies for being published in its present form.
Response: We thank the reviewer for their acceptance of our prior response. There are no additional comments to address.

Reviewer Comment #2: I have read with great interest the article "Real time assesment of vascular occlusion and reperfusion in animal models of intraoperative imaging" which deals with the establishment of "SurgeON BFN" in a large animal model. The colleagues have already been able to publish their preliminary data at a high level (Sci Rep 2020) and therefore have visible expertise in laser speckle contrast imaging (LSCI). The clinical need for dynamic methods to assess organ perfusion in real time is unquestionable. The advantages of LSCI compared to ICG and conventional Doppler diagnostics have been demonstrated and discussed. The graphs are clear and facilitate the understanding of the study. As already criticized by the pre-reviewers, the Rabbit model is not ideal for the application of LSCI especially in comparison to ICG especially since the possibilities for the application of LSCI (transplantation, intestinal anastomoses etc.) seem almost unlimited.
Overall, this is a pilot study which should perhaps be mentioned in the title, since the results of the study are limited and above all the feasibility is demonstrated. Furthermore, the colleagues should mention in the discussion which concrete further steps are necessary to make LSCI clinically applicable.
Response: Appropriate changes have been added to the manuscript and highlighter in gray. Specifically,
1) The title has been modified to:
REALTIME ASSESSMENT OF VASCULAR OCCLUSION AND REPERFUSION IN ANIMAL MODELS OF INTRAOPERATIVE IMAGING: PILOT STUDY
2) A clarification regarding the pilot nature of the study and the need for additional development prior to clinical translation has been included in the Discussion section (last paragraph of the section).

We hope the reviewers will find our response satisfactory and adequate.

Comments by the Editor-in-Chief Decision

All reviewer comments were addressed adequately and the manuscript should be published in its present stage.
